# Direct correlation analysis improves fold recognition

**DOI:** 10.1016/j.compbiolchem.2011.08.002

**Published:** 2011-10-12

**Authors:** Michael I. Sadowski, Katarzyna Maksimiak, William R. Taylor

**Affiliations:** Division of Mathematical Biology, MRC National Institute for Medical Research, The Ridgeway, Mill Hill, London NW7 1AA, UK

**Keywords:** Protein fold recognition, Decoy models, Direct information

## Abstract

The extraction of correlated mutations through the method of direct information (DI) provides predicted contact residue pairs that can be used to constrain the three dimensional structures of proteins. We apply this method to a large set of decoy protein folds consisting of many thousand well-constructed models, only tens of which have the correct fold. We find that DI is able to greatly improve the ranking of the true (native) fold but others still remain high scoring that would be difficult to discard due to small shifts in the core beta sheets.

## Graphical abstract

**Graphical abstract:**

**Figure:**
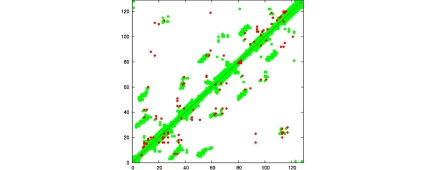

**Highlights:**

► The problem of protein prediction from sequence is difficult and incompletely solved. ► We show that a new method based on correlated mutations in a multiple sequence alignment, filtered through a process to extract direct contacts provide powerful constraints on selecting the correct fold in a large number of well constructed decoy models.

## 1 Introduction

For many years it has been thought that physical interaction between residues in a protein structure would create constraints on mutation that would then be apparent in an evolutionary analysis of a multiple sequence alignment. The analysis of coordinated changes across a multiple sequence alignment might then be used to detect interacting residues from which spatial proximity could be inferred (Altschuh et al., 1987; Hatrick and Taylor, 1994; Neher, 1994; Gobel et al., 1994). Since these early attempts, there have been many additional improvements and new approaches, including consideration of phylogenetic effects (Pollock and Taylor, 1997; Pollock et al., 1999; Wang et al., 2006) and structural effects (Singer et al., 2002; Valencia and Pazos, 2002) and also both together (Lapedes et al., 1999). Almost always these methods produced inconclusive results, and none were powerful enough to generate constraints that would allow the specification of a 3D protein structure, with the limitation usually being identified as insufficient sequence data.

A more recent attempt using the method of statistical coupling analysis (SCA) brought revived interest through promising results combined with experimentation (Lockless and Ranganathan, 1999; Suel et al., 2003; Socolich et al., 2005) and this approach produced encouraging results when combined with a method that generated realistic structural models (Bartlett and Taylor, 2007). Using models derived from an orthogonal source avoided the need to rely on the quality of the predicted contact to construct a model using distance geometry as the sets of distances had only to be evaluated, not created by the correlation signal. This made it less critical to determine whether the correlated signal resulted from a direct contact or from an indirect effect in which triples or chains of residues were co-evolving, a known confounding effect for methods based on standard estimates of correlation such as mutual information (MI).

In the more general case of solving a structure using predicted contacts such indirect correlations cause significant problems and lead to insoluble constraints on the system, creating a need to tease apart the direct correlations from the indirect correlations. This is a difficult problem and although the statistical framework to deal with it had been established some time ago (Lapedes et al., 1999) it had largely been ignored until recently (Weigt et al., 2009; Burger and van Nimwegen, 2010). The method of Weigt and co-workers was primarily focused on the interaction of two proteins but besides the inter-molecular interactions, the intra-molecular interactions are also identified. For some small members of the chemotaxis Y family (cheY), these appeared to be sufficient to identify the correct fold from a collection of decoys but probably not strong enough for direct calculation of a unique fold by distance geometry. On a larger family of Ras proteins (Weigt, 2011), there is a very clear intra-molecular signal which could prove to be very powerful in fold recognition.

In this work, we use a of collection of model or “decoy” folds, similar to those tested previously with the SCA method, to evaluate the power of direct correlation analysis (DCA) at ranking the folds and discriminating the native fold from a very large multitude of well constructed decoys.

## 2 Results and discussion

### 2.1 Generation of decoy models

Decoy models were generated completely automatically using our previously published methods (Taylor et al., 2008, 2009) that are implemented as the server PLATO which was used to make predictions for the recent CASP-9 exercise.

This method takes only a single sequence and compiles a multiple alignment (filtered to remove redundancy) that is used to predict secondary structures with the PSIPRED method (Jones, 2000). However, to avoid any bias towards known structures, the PSIPRED sequence database is composed only of sequences from the aligned family of the protein being predicted.

As described in more detail in Section 4, the resulting secondary structures are mapped onto all idealised frameworks (Forms) that can support them and the chain paths are enumerated combinatorially to generate a wide variety of different folds. Each fold is elaborated into an alpha carbon model that is evaluated both by standard measures of protein structure and in the current work, by the predicted contact data from direct correlation information (DCA) (see Section 4 for details).

As previously (Bartlett and Taylor, 2007), we consider a collection of proteins drawn from the three-layer αβα architecture and focus principally on two proteins for which there is also published data. One of these is of moderate size (128 residues) representing the cheY-family (3chy) (Weigt et al., 2009) while the other is a larger protein with 166 residues representing the ras-family (5p21) (Weigt, 2011). CheY had been used previously in a study of correlated changes in sequence using the statistical coupling analysis (SCA) method (Lockless and Ranganathan, 1999) and had performed well using those data (Bartlett and Taylor, 2007). However it was not the best protein used in that study with two others attaining better results and two worse. The ras (p21) protein, although only slightly larger, results in many more possible folds (because of the combinatoric nature of the model generating algorithm). This combined with an unusual connection of the more N-terminal edge of the domain makes it a difficult target.

The cheY-family alignment gave rise to 8567 models which contained 1216 different folds as distinguished by their secondary structure topology strings (see Section 4). As these are unique, only one string corresponds to the correct fold although sometimes it is reasonable to consider minor deviations from this. Taking the strictest definition, the total set of 8567 folds contained only 23 correct folds which have the topology string:

+B+0.−A+0.+B−1.−a+0.+B+1.−a+1.+B+2.−a+2.+B+3.−A+1.

These constituted the true matches that were used to calculate the receiver–operator curves (ROC) that we used to characterise the success of each scoring scheme below. The basic scoring scheme used to rank the full set of folds is based on a combination of simple physico-chemical properties (Taylor et al., 2006). In the PLATO protocol, this measure is used to reduce the full set prior to a more detailed evaluation which with the current cheY-family resulted in a reduced selection of 1332 folds containing 16 true folds: an enrichment of over four fold from 0.27% to 1.2%. This re-scoring lifted the first occurrence of the true fold from position 30 to 1.

The longer ras sequence (166 residues) generated almost double the number of models as cheY (15623) on the initial stage of PLATO, containing 4164 distinct folds. These were reduced to 2372 models (637 folds) after re-scoring and filtering to produce the final ranked list. In the initial (full) list, there were 20 correct folds and 12 in the final (best) list with topology string:

+B+0.−A+0.−B−2.+B−1.−a+0.+B+1.−a+1.+B+2.−a+2.+B+3.−A+1.

Because of the unusual topological arrangement on the amino terminal edge of the domain, comprising a βα-unit linked to a β-hairpin by a parallel connection (+B+0.-A+0.-B-2.+B-1), the true folds were ranked much lower in these lists with the top fold at rank 247 in the full list and 38 in the re-ranked (best) list. This puts the correct answers well below what would be considered for full molecular refinement and energy calculation (or serious consideration in the CASP exercise).

### 2.2 CheY-family model re-ranking

**Fig. 1:**
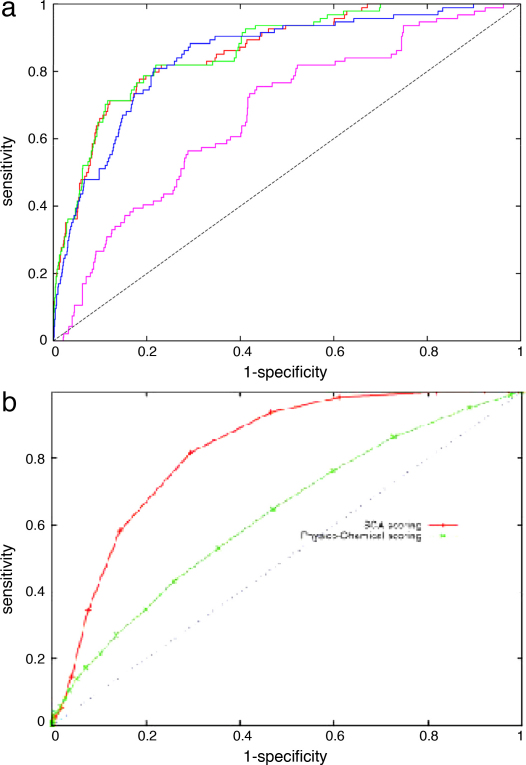
ROC plots for CHEY based on the frequency of occurrence of the true fold in the ranked list of models (see text for details). In part a, the purple curve is based on the raw PLATO scores where as the other curves are increasingly weighted by DCA contact data in the order: blue < green < red, with the green curve being close to equal weighting. Part b shows the corresponding data from (Bartlett and Taylor, 2007) with the raw physico-chemical scored folds plotted in green and the SCA weighted ranking in red. (For interpretation of the references to color in this figure legend, the reader is referred to the web version of the article.)

Reproduced with permission.

Introducing the contribution from the pairs of residues identified from the direct correlation analysis made a powerful positive contribution to the re-ranked order. The correct fold retained its top position and many more true folds were encountered higher in the ranking. This can be seen in the ROC plot Fig. 1 as a shift in the curves towards the upper left corner from the ranking based only on the PLATO score (purple) to those with a increasing contribution from the DCA score. Although the difference in these latter curves is small, a slightly better performance is obtained with roughly an equal contribution from DCA and PLATO scores (green).

**Fig. 2:**
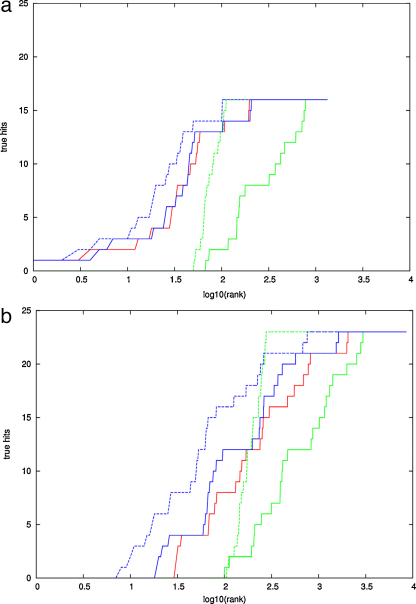
Log ‘ROC’ plots for CHEY. The cumulated number of true folds (*Y*-axis) is plotted against the log value of the position in the ranked fold data: a for the final (best) and b for the initial (full) number of folds generated by PLATO. On each plot, green is the ranking for the DCA contacts alone, red is the raw PLATO score and blue their combined score. Dashed lines are calculated from the data of Weigt et al. (For interpretation of the references to color in this figure legend, the reader is referred to the web version of the article.)

While good, this result is not a dramatic improvement over the shift in curve obtained previously (Bartlett and Taylor, 2007) using the statistical coupling analysis (SCA) method which does not incorporate any evaluation of DCA. However, for this type of comparison the ROC plots are not ideal as the *X*-axis represents the complete ranking of 8000+ folds and the ones we are primarily interested in is the extent to which the 20-odd true folds are found near the origin. In this region, it can be noted that there is a distinct shift of the contact weighted curves towards the *Y*-axis, indicating that more true folds have a better ranking than with SCA. To expand this region, we simply plotted the raw ranked data as log(rank) along the *X*-axis and the number of true folds on *Y* (Fig. 2).

The numbers of true hits are plotted for final reduced list of PLATO models (Fig. 2) when ranked by their PLATO score (red), the DCA score (green) and the combined PLATO–DCA score (blue) which is slightly better than either individually. On the full list of models, however, the shift in ranking is more dramatic with the top true fold rising from 30 to position 9 with the combined score.

**Fig. 3:**
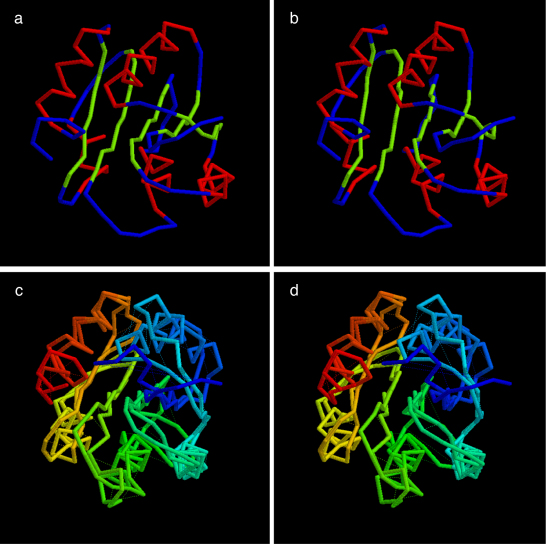
Top scoring true fold for CHEY as identified in the final (best) PLATO ranking by the combined physico-chemical and DCA score. Parts a + b constitute a stereo pair of the alpha-carbon trace coloured by secondary structure type (α = red, β = green). Parts c + d are also a stereo pair of the same fold superposed on the native structure (3chy) and coloured from amino (blue) to carboxy (red) for both structures. (For interpretation of the references to color in this figure legend, the reader is referred to the web version of the article.)

**Fig. 4:**
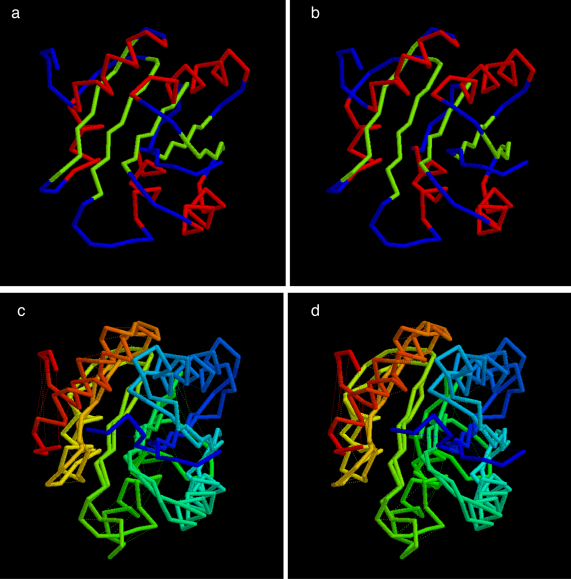
Top scoring true fold for CHEY as identified in the initial (full) PLATO ranking by the combined physico-chemical and DCA score. The parts are as in Fig. 3.

The quality of the top ranked true models is typical of that reported previously (Taylor et al., 2008) with RMS deviation from the native structure of 4.6/125 (Å/res.) for the refined PLATO ranking (Fig. 3) and 4.0/120 (Å/res). for the full list of folds (Fig. 4).

Besides the rank and quality of the true models, it is informative to examine the nature of the folds that are in competition with the true fold. This can be easily seen from the lists of ranked topology strings for both the final (left) and initial (right) PLATO + DCA rankings (where a ‘*’ marks correct folds):

**Figure:**
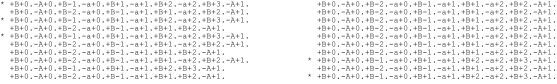

The dominant fold that shadows and often betters the true fold is very similar and has only a single exchange of two positions with the initial β-strand moving to the central position in the five stranded sheet:

+B+0.−A+0.+B−2.−a+0.+B−1.−a+1.+B+1.−a+2.+B+2.−A+1.

**Fig. 5:**
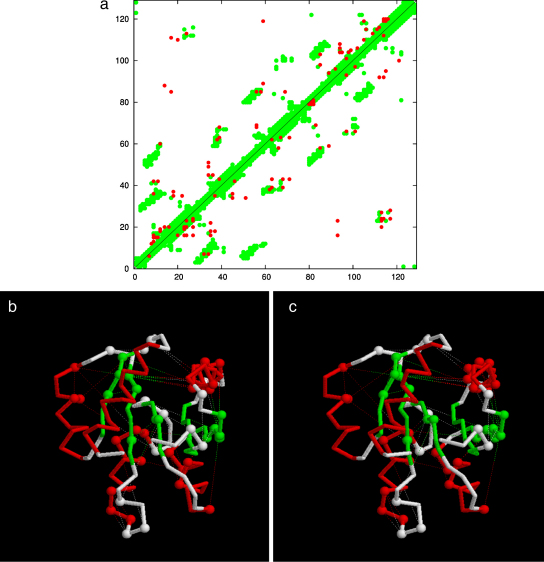
Distance and DCA plot for CHEY. (a) The contact map for 3chy is plotted for residue pairs closer than 8 Å (green), over which the residue pairs identified by DCA are plotted in red with the current method in the top-left and the Weigt et al. method lower-right. Parts b + c are a stereo pair showing the residues selected by DCA as spheres with fine lines linking pairs. (For interpretation of the references to color in this figure legend, the reader is referred to the web version of the article.)

It can be seen from the distance plot of the DCA that this is not a strongly constrained region as the first strand (lower left corner) has only a few predicted contacts with its consecutive helix and none with other β-strands (Fig. 5). Even with much better constraints, it is likely that such errors would still arise as they can be seen even when RMS-based superposition is used to select model structures (Hollup et al., 2011).

### 2.3 Ras-family model re-ranking

**Fig. 6:**
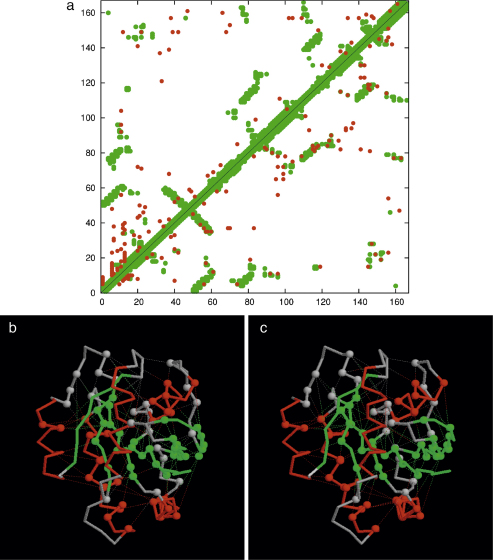
Distance and DCA plot for Ras (plotted as in Fig. 5). (For interpretation of the references to color in this figure legend, the reader is referred to the web version of the article.)

**Fig. 7:**
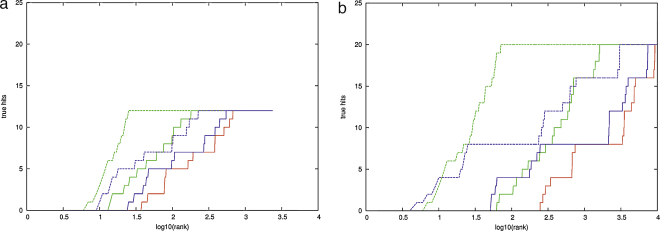
Log ‘ROC’ plots for Ras (plotted as in Fig. 2). (For interpretation of the references to color in this figure legend, the reader is referred to the web version of the article.)

The protocols and analysis described above for the cheY family were repeated on the larger ras (p21) family using an unbiased automatic prediction of model structures by PLATO and an equivalent density of contacts predicted by direct information (DCA). As can be seen from the contact map (Fig. 6) the ras family contains many well distributed correct contacts and the ROC plot based on the re-ranked list identified all the correct folds under a level of 0.01 (1-sensitivity). As such a plot is not visually informative, we plotted the raw data against log(rank) as described above (Fig. 7).

It can be seen from these plots, that the contribution of DCA lifted the rank of the top true fold into the top 10 in both the initial and final PLATO fold lists with the contribution from the PLATO scores having less effect. These produce a slight improvement of the top rank in the full list to fifth place (Fig. 7b) but have a correspondingly slight detrimental effect on the final PLATO list (Fig. 7a).

**Fig. 8:**
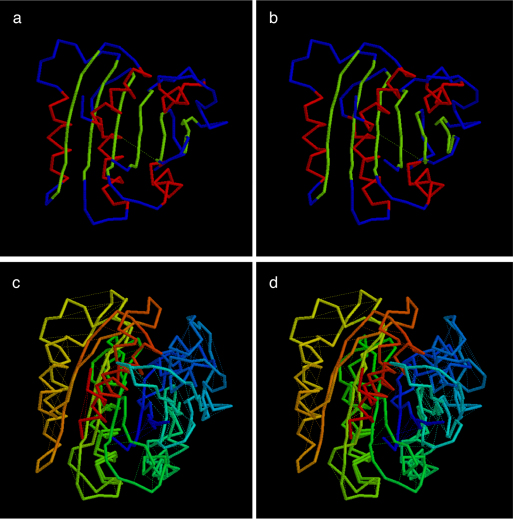
Top scoring true fold for Ras (plotted as in Fig. 3). (For interpretation of the references to color in this figure legend, the reader is referred to the web version of the article.)

The resulting models corresponding to the top true hits have a worse RMS deviation from the native structure relative to the cheY family, even allowing for the larger size of the Ras structure. This comes mostly from variations in the long β-hairpin on the amino terminal edge of the domain discussed above. The fold which is ranked top in both lists has an RMS of 7.5/157 (Å/res.) but taking a smaller subset of residues, that excludes some of the problematic hairpin, reduces this to 5.5/100 which is more typical for a domain of this size (Taylor et al., 2008). The model is shown in Fig. 8 along with its comparison to the native.

As with CHEY, it is informative to look at the folds that are in competition with the true fold as these can reveal weaknesses in the constraints. The top ten ranked folds for each list are shown as above (’*’ = true):

**Figure:**
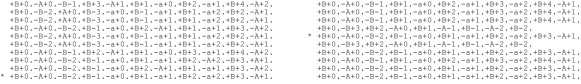

**Fig. 9:**
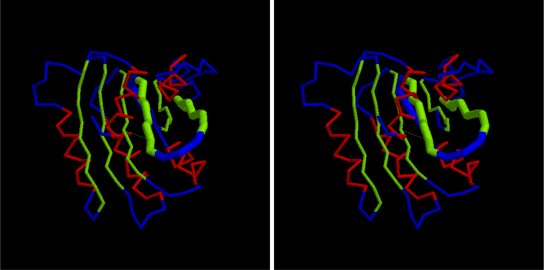
Top scoring fold for Ras with the region that differs from the native emphasised as a thicker trace. This change is not apparent from the overall RMSD value which is lower than that obtained with the correct topology (plotted as in Fig. 3). (For interpretation of the references to color in this figure legend, the reader is referred to the web version of the article.)

There is a wider variety of fold variants ranked higher than the true fold than was seen for the cheY models but these are again dominated by variations in which β-strands have swapped positions in the sheet, especially in the problematic hairpin region on the N-terminal end of the domain. The top scoring fold in both lists has the edge hairpin split either side of the initial strand (Fig. 9) but is otherwise correct. Again this corresponds to a region of sparse contacts in the data. Interestingly, this model has a better superposition on the native structure than the top true fold with an RMS of 5.0/116 (Å/res.), confirming the observation that any RMS based measure is not ideal for evaluating topological correspondence (Hollup et al., 2011).

### 2.4 Summary of results for all proteins

In addition to the two proteins considered in detail above, the method was run automatically over the other proteins included in the analysis of Bartlett and Taylor (2007) with the exception of the protein with PDB code: 1di0, as this protein is strongly multimeric and does not have a large sequence family.

In Table 1, it can be seen that for every protein considered, the inclusion of DCA contact information has resulted in an improvement in the rank of the top true fold.

## 3 Conclusions

We have shown in this study that direct contact information extracted from an analysis of residue covariation provides a powerful contribution to improving the ranking of the true fold in a large collection of well-formed decoy folds. The examples considered share a similar architecture but have different folds and have in the past presented different challenges to an *ab initio* prediction approach. cheY is compact and regular with well formed secondary structure elements that obey standard packing rules. At almost 130 residues, the cheY structure would be considered to be at, or beyond, the limit of anything that could be predicted without structural information. With almost 40 more residues, the Ras structure would certainly be beyond all folding methods and with some unusual packing arrangements (discussed above) presents a difficult challenge for prediction.

Applied to the cheY derived decoys, the DCA scores made a marked improvement in the ranks of the true folds. This was better than previous results using the SCA method, but not dramatically so. The covering of the contacts was uneven, allowing considerable freedom in some secondary structure elements (SSE) to adopt alternative positions and still score well. This was particularly deleterious in the case of the first β-strand which can adopt an alternative position in the sheet that still preserves its few contacts and buried hydrophobic environment. Similarly, if there are only a few constraints all to the same region of a SSE, then both orientations of the SSE will be equally favoured. This was seen mostly in the terminal α-helix which, of course, is less well ‘tied-down’ by its chain connections. As determined previously in the context of distance geometry applications, the minimal ideal distribution of constraints is to have them covering both ends of every SSE (Aszódi et al., 1995).

The better quality of the constraints on the Ras structure produced a dramatic improvement in the ranking of the true folds, bringing them within the top 10 and to rank 5 in the best situation—close enough to be selected as a solution for the CASP experiment. Despite the high quality of the Ras data, alternative fold solutions still remained high scoring, especially when the constraints involved β-strand positions in the sheet. Without constraints to adjacent strands, the swapping of strands to adjacent positions in the sheet is effectively undetectable over longer ranges. For example in the Ras models, the edge β-hairpin was split by the N-terminal strand. The constraints on these positions were imposed mainly from the helices that pack either side but from this position, the distances in the alternative folds are similar.

The use of direct information can be very powerful and in our test examples, comes close to bringing the correct fold within a sufficiently small number of alternatives that could be evaluated using more computationally expensive methods based on all-atom refined folds. In this work we wanted only to test the power of the method at the fold-recognition level based on simple scoring measures. However, it was of interest to see if the data were sufficient to generate a unique (or any) structure using distance geometry but using the DRAGON program (Aszódi and Taylor, 1994) this was unsuccessful, suggesting that the use of pre-formed models derived from ideal folds provides an important contribution.

## 4 Methods

### 4.1 Model generation using PLATO

A server for *ab initio* protein structure prediction using ideal forms (Taylor, 2002) was developed. This was essentially a fully automated version of the previously described build method (Taylor et al., 2008), however it included refinements to enable a fully automatic solution and improve model selection and ranking.

Profiles for target sequences were generated by alignment to a local copy of the NR database using PSIBLAST (Altschul et al., 1997) following which the alignment was culled to a small number of representatives. Predictions of secondary structure were made using two methods (PSIPRED, Jones, 1999, Jones, 2000 and YASPIN, Lin et al., 2005) for all representatives in the alignment. Predictions were grouped and converted to element-level predictions by testing all possible alternatives for ambiguous elements: present/absent for short elements (less than 3 for strands, less than 4 for helices) and helix/strand for ambiguous regions.

For each prediction, all compatible ideal forms were identified and used as templates for prediction. A given form provides a lattice representation for an arrangement of secondary structures in either a three-layer α/β/α, four-layer α/β/β/α or polyhedral all-α arrangement. Possible topologies were generated for each lattice by generating all permutations compatible with lengths of predicted loops and sequence hydrophobicity. In a novel step the choice of lattice was filtered by comparison with known SSE sequences using BLAST. A population of thousands of α-carbon models was generated in this way for each domain.

All generated models were based only on α-carbon positions and full atomic models were not used generated in this work but can be automatically constructed from the α-carbon positions with good accuracy (MacDonald et al., 2009).

### 4.2 Topology string encoding and matching

**Fig. 10:**
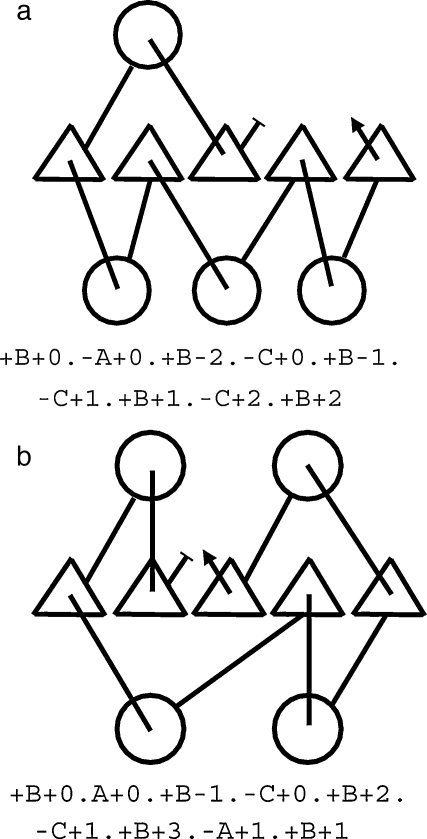
Example topology strings. Two small αβα layer proteins fitting form 1–5–3 (a) (one helix above and three below a five-stranded sheet) and form 2-5-2 (b) are shown as topology diagrams with their corresponding topology strings below. In the topology diagrams, helices are depicted as circles and β-strands as triangles. In the topology strings, the three layers of secondary structure (αβα) are designated A (top), B and C, respectively. Each SSE is given a label of three parts indicating orientation (“ + ”, “−”), layer and position in the layer. The first SSE in each layer is, by definition, at position 0 with others numbered relative to this. In the topology diagram negative numbers lie to the left, positive to the right. Similarly, in the strings, a positive orientation corresponds to a SSE approaching (“out of the page”) in the diagrams.

The folds for the model structures were encoded as strings that specify the coordinates of the chain through the lattice (Form). Each match to a Form allows the fold to be specified by its path over the underlying lattice. This can be done in a simple coordinate system which uses the letters “A”, “B” and “C” (or “a” if the layer is α) for the three layers, and a number for position in the layer with the remaining dimension requiring only two values, “+” or “−”, to designate front and back. The first SSE to enter a layer is assigned position 0 and the first strand in the sheet takes the positive orientation, giving “+B+0” in the string. The first α-helix then sets the top/bottom orientation by assigning its layer as “A”. The resulting strings (referred to as “topology strings”) are quite easy to read and visualise the fold. Two examples are given in Fig. 10.

### 4.3 Contact calculation and model evaluation

As previously, residue contacts were predicted from pfam multiple sequence alignments (Bartlett and Taylor, 2007). Staring from a standard calculation of mutual information between alignment positions (Weigt et al., 2009), these values were normalised using a heuristic implementation of the direct information algorithm (Lapedes et al., 1999) and renormalised to improve consistency with expected cumulative residue packing distributions (Aszódi and Taylor, 1995). Residue contact pairs calculated by the direct information algorithm of Weigt et al. (2009) were taken from supplementary information for the cheY family and for the ras family from Weigt (2011) which were kindly verified by the author.

The models were assessed by summing the distance between pairs of residues over the contact list, resulting in a value (*D*) that should be as small as possible. As the PLATO scores (*P*) are larger for good models, the two scores were combined as *P*/*D* making the combination scale insensitive.

## Figures and Tables

**Table 1:** Fold recognition over five decoy sets. For the five proteins considered (PDB) each decoy in the PLATO selected set (best) and complete set (full) was ranked by the basic PLATO scoring scheme (base) and by the DCA augmented combined score (comb). The ranked positions are the first occurrence of the true fold in the list (with the position counting only unique folds in parentheses). The root mean square deviation (RMSD) value is calculated over the number of residues shown in parentheses for the top model in the combined ranking of the best PLATO set. These values are typical although the 2trx variant selected had a short helix/loop in an exposed configuration giving a higher than average value for this fold.

PDB code	Number of decoy models		True fold rank (base)		True fold rank (comb)		Comb best top model
	Best	Full	Best	Full	Best	Full	RMSD (Å/res)
2trx	4397	29647	9(6)	23(6)	7(4)	22(10)	6.17/100
1coz	2822	21052	15(11)	55(18)	13(10)	30(10)	5.42/109
3chy	1332	8567	1(1)	30(9)	1(1)	19(4)	4.62/125
1f4p	4243	25676	68(37)	20(12)	57(31)	7(6)	6.32/141
5p21	2372	15623	38(21)	247(98)	25(15)	52(24)	6.25/156
